# Supplementary figures and images for: The Effectiveness of Glutathione Redox Status as a Possible Tumor Marker in Colorectal Cancer
Source: Int J Mol Sci. 2021 Jun 8;22(12):6183. doi: 10.3390/ijms22126183 (PMC8226858; doi:10.3390/ijms22126183)

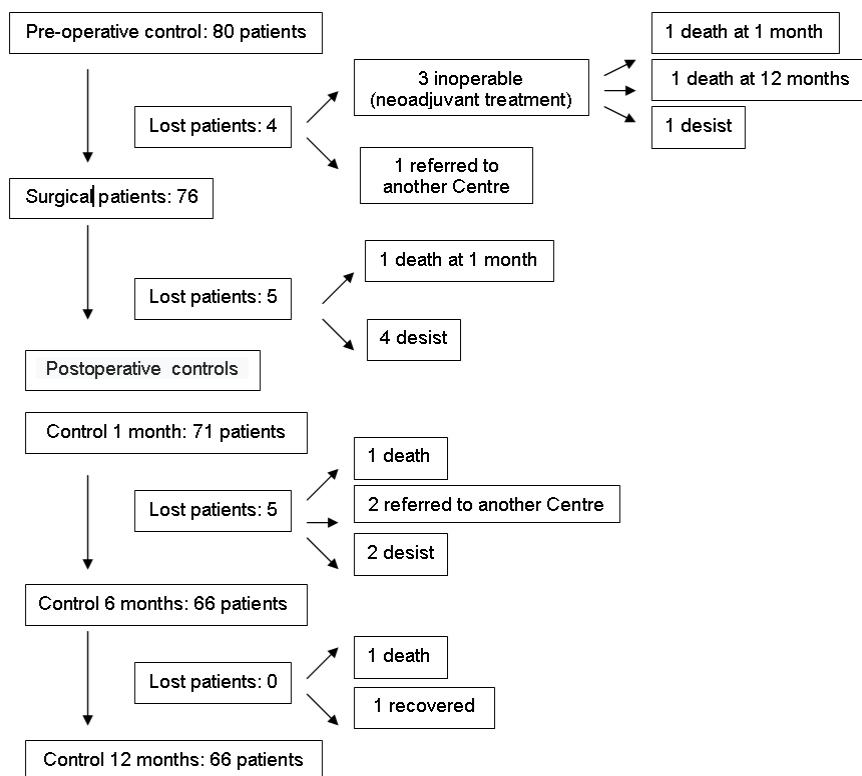

Supplement: Supplementary file 1 [file ijms-22-06183-s001.zip › ijms-1219530-supplementary.pdf]
